# Parathyroid Hormone Mediates Hematopoietic Cell Expansion through Interleukin-6

**DOI:** 10.1371/journal.pone.0013657

**Published:** 2010-10-27

**Authors:** Flavia Q. Pirih, Megan N. Michalski, Sun W. Cho, Amy J. Koh, Janice E. Berry, Eduardo Ghaname, Pachiyappan Kamarajan, Edith Bonnelye, Charles W. Ross, Yvonne L. Kapila, Pierre Jurdic, Laurie K. McCauley

**Affiliations:** 1 University of Michigan School of Dentistry, Ann Arbor, Michigan, United States of America; 2 Institut de Génomique Fonctionnelle de Lyon, Université de Lyon, Université Lyon 1, CNRS, INRA, Ecole Normale Supérieure de Lyon, Lyon, France; 3 Department of Pathology, University of Michigan, Ann Arbor, Michigan, United States of America; 4 Faculté de Médecine RTH Laennec, Lyon, France; New York University, United States of America

## Abstract

Parathyroid hormone (PTH) stimulates hematopoietic cells through mechanisms of action that remain elusive. Interleukin-6 (IL-6) is upregulated by PTH and stimulates hematopoiesis. The purpose of this investigation was to identify actions of PTH and IL-6 in hematopoietic cell expansion. Bone marrow cultures from C57B6 mice were treated with fms-like tyrosine kinase-3 ligand (Flt-3L), PTH, Flt-3L plus PTH, or vehicle control. Flt-3L alone increased adherent and non-adherent cells. PTH did not directly impact hematopoietic or osteoclastic cells but acted in concert with Flt-3L to further increase cell numbers. Flt-3L alone stimulated proliferation, while PTH combined with Flt-3L decreased apoptosis. Flt-3L increased blasts early in culture, and later increased CD45^+^ and CD11b^+^ cells. In parallel experiments, IL-6 acted additively with Flt-3L to increase cell numbers and IL-6-deficient bone marrow cultures (compared to wildtype controls) but failed to amplify in response to Flt-3L and PTH, suggesting that IL-6 mediated the PTH effect. *In vivo*, PTH increased Lin^-^ Sca-1^+^c-Kit^+^ (LSK) hematopoietic progenitor cells after PTH treatment in wildtype mice, but failed to increase LSKs in IL-6-deficient mice. In conclusion, PTH acts with Flt-3L to maintain hematopoietic cells by limiting apoptosis. IL-6 is a critical mediator of bone marrow cell expansion and is responsible for PTH actions in hematopoietic cell expansion.

## Introduction

Parathyroid hormone (PTH) and parathyroid hormone related protein are pleiotropic factors that operate via endocrine, paracrine, autocrine and intracrine modes of action. They are implicated in different processes such as epithelial-mesenchymal interactions, skeletogenesis and carcinogenesis [1], [2], [3]. PTH has a positive impact on hematopoietic stem cells (HSCs), and is currently being investigated as a potential therapeutic to stimulate hematopoiesis and enhance bone marrow engraftment [4], [5], [6]. Despite extensive research on PTH skeletal actions, the mechanisms for the hematopoietic impact are still elusive.

Both direct and indirect actions of PTH on cells of the hematopoietic lineage have been proposed. PTH has long been known to activate osteoclasts, cells of hematopoietic origin formed by the differentiation and fusion of mononuclear monocyte-macrophage lineage precursors that are responsible for bone resorption. This activation is widely accepted to be indirect via an upregulation of RANK-L in cells of the osteoblast lineage [7], however reports exist of PTH receptors in osteoclasts as well [8], [9]. The anabolic actions of PTH in bone have been suggested to be associated with the differentiation stage of cells in the osteoclast lineage [10]. Furthermore, other hematopoietic cells have been proposed as targets of PTH action. T-lymphocytes have PTH receptors and PTH induces altered responses in a T-cell deficient background [11], [12], [13]. Many unanswered questions persist regarding the impact of PTH on the variety of cells occupying the bone marrow microenvironment.

PTH regulates several genes associated with hematopoiesis including interleukin-6 (IL-6) [14]. IL-6 is a multifunctional cytokine with diverse effects ranging from cell proliferation and differentiation to apoptosis and cell survival [15]. IL-6 stimulates proliferation of early hematopoietic progenitor cells (HPCs) [16]. IL-6 null mice are apparently normal in terms of their survival, development, skeletal phenotype and response to catabolic PTH [17]. Interestingly, IL-6 deficient mice have decreased numbers of HPCs, defective liver regeneration and altered susceptibility to arthritis [18].

The purpose of this study was to determine the mechanism by which PTH acts on cells of the hematopoietic lineage. The central hypothesis is that PTH acts on bone marrow stromal cells to stimulate IL-6 production. IL-6 in turn synergizes with *fms*-like tyrosine kinase 3 ligand (Flt-3L), to increase hematopoietic cell numbers. Given that IL-6 is upregulated by PTH and is also a regulator of hematopoietic stem cells, the PTH induction of IL-6 in stromal cells and its additive effects with Flt-3L results in hematopoietic cell expansion. We conclude that PTH increases hematopoietic cells *ex vivo* via an inhibition of apoptosis in Flt-3L responsive cells. PTH indirectly increases hematopoietic progenitor cells and does not directly affect osteoclast lineage cells. Stromal cell derived IL-6 in conjunction with Flt-3L mediates the PTH activation of hematopoietic cells.

## Materials and Methods

### Mice

All experimental animal procedures were performed in compliance with institutional ethical requirements and approved by the University of Michigan Committee for the Use and Care of Animals. Wild-type (Jackson Laboratory, Bar Harbor, ME, USA) and IL-6 deficient [19] (kindly provided by Evan Keller, University of Michigan, Ann Arbor, MI, USA) C57B6 mice at 4–8 wks of age were used for *ex vivo* experiments. For *in vivo* experiments, mice received subcutaneous injections of vehicle (saline) or 50 µg/kg/day human PTH (hPTH 1–34, Bachem; Torrance, CA, USA) for three weeks beginning at 4 days of age, then were sacrificed 48 hours after the last injection as previously described [20].

### Bone marrow cell isolation and *ex-vivo* cell amplification

Total bone marrow cells from femurs and tibiae were isolated by extraction of long bone marrow into Iscove's Modified Dulbecco's Medium (IMDM; Invitrogen; Carlsbad, CA, USA) followed by filtration through a nylon mesh screen (70 µm, BD Falcon, Franklin Lakes, NJ, USA). The *ex vivo* amplification protocol is based on Servet-Delprat's model system [21]. In brief, cells were seeded at 1.8×10^5^/cm^2^ in IMDM supplemented with 20% fetal bovine serum, 100 units/ml penicillin, 50 µg/ml streptomycin and 1% glutamine. At the time of plating, cells were treated once with Flt-3L (5 ng/ml or 100 ng/ml. Although 2 different concentrations were utilized, they produced the same biological effect) (Emory University; Atlanta, GA, USA or R&D Systems; Minneapolis, MN, USA) and/or PTH 10 nM. *Ex vivo* experiments were performed on day 8 of culture unless otherwise specified. Cells were enumerated using a hemocytometer on days 2, 4, 6 and 8 and cell viability was determined by trypan blue dye exclusion. In similar experiments, C57B6 bone marrow cells were harvested and treated with 10 ng/ml mIL-6 (R&D Systems) plus vehicle, PTH, Flt-3L, PTH with Ftl-3L and/or the addition one hour later of cucurbitacin, a STAT inhibitor, (20 and 40 nM) (Calbiochem, San Diego, CA, USA).

### RNA extraction and quantitative reverse transcriptase-polymerase chain reaction

Total RNA was collected from non-adherent cells at days 2, 4, 6 and 8 and from fresh bone marrow (used as a positive control). RNA isolation was performed using Trizol reagent (Invitrogen) according to the manufacturer's protocol. Total RNA (0.5 µg) was reverse transcribed using TaqMan® Reverse Transcription Reagents (Applied Biosystems; Branchburg, NJ, USA) according to the manufacturer's protocol. One microliter (1 ng) of reverse transcribed product was amplified with TaqMan® Universal PCR Master mix (Applied Biosystems) and gene-specific primers designed by Applied Biosystems (Flt3 Mm00438996_m1 and GAPDH 4308313). The amplification program was set for 1 cycle at 50°C for 2 min, 1 cycle at 10°C for 10 min followed by 40 cycles at 95°C, 15 sec; 60°C, 1 min using the Applied Biosystems 7500 Real-Time PCR System. Relative induction was determined by the 2^-ΔΔCt^ method using GAPDH and the fresh bone marrow extraction for normalization and comparison [22].

### Flow cytometric analyses

Flow cytometric analyses (FACS) of bone marrow extractions and cultured cells were performed. For the *in vivo* experiments bone marrow cells were isolated as described above, rinsed and resuspended in cold FACS buffer (PBS supplemented with 2 mM EDTA and 1% FBS). A small aliquot was treated with 1X ACK Lysis Buffer and enumerated without red blood cells. Cells (5×10^6^/sample) were incubated for 45–60 minutes at 4°C with appropriate antibodies and protected from light exposure. Cells were washed with FACS buffer, resuspended and analyzed on a FACSCalibur (BD Biosciences, San Jose, CA) using the Cellquest-Pro software (BD Biosciences) to detect specific cell populations. All antibodies were acquired from BD-Pharmingen and the cell markers analyzed were Ly-6A/E (Sca1^+^), CD117 (c-Kit^+^, 2B8), CD45R/B220, and Ly-6g and Ly-6C (Lineage-). To analyze apoptosis the BD-Pharmingen AnnexinV: FITC conjugated apoptosis assay system was used, following the manufacturer's protocol.

E*x vivo* experiments were performed to characterize cell populations, apoptosis and cell cycle. To identify different cell populations, non-adherent cells were collected at various time points, pelleted, rinsed with PBS and then incubated with the appropriate antibody. The following antibodies were utilized: IL7Rα^+^, CD19^−^, CD3^−^ (lymphoid progenitor cell) CD45^+^ (cells of the hematopoietic lineage excluding erythrocytes), CD11b^+^ (monocyte/macrophage), GR-1^+^ (granulocyte), CD3^+^ (T-cell) and CD-19^+^ (B-cell) antibody (BD Biosciences). To analyze apoptosis the BD-Pharmingen AnnexinV: FITC conjugated apoptosis assay system was used. Samples were run using the FACSCalibur system and data was analyzed with Cell Quest Pro software. For cell cycle analysis cells were fixed in 50% cold ethanol, pelleted then stained with 10 µg/ml Propidium Iodide (BD Biosciences) and 100 µg/ml RNAse (Sigma-Aldrich, St. Louis, MO, USA). Data were acquired using a FACSCalibur system and data analyzed with ModFit software (Verity Software House, Topsham, ME, USA).

### Protein extraction and western analysis

Suspension cells underwent centrifugation and were washed once with PBS, then resuspended in CelLytic MT mammalian tissue lysis extraction reagent (Sigma-Aldrich) with 1% Protease Inhibitor Cocktail (Sigma-Aldrich). After incubation, supernatants were collected for analysis. SDS-PAGE was performed on 4–20% gradient acrylamide gels, loading 30 µg/sample. Membranes were blocked for 1 hour in 5% nonfat milk in TBST, incubated overnight with cyclin D1 antibody (Cell Signaling Technology, Danvers, MA, USA), rinsed with TBST and incubated with secondary antibody (GE Healthcare, Piscataway, NJ, USA). After rinsing with TBST, membranes were incubated with enhanced chemiluminescence reagents (Pierce Biotechnology; Rockford, IL, USA) and exposed to BioMax film. Bands were normalized with actin, and compared using either ImageJ analysis program (Wayne Rasband, wayne@codon.nih.gov) or a Chemidoc visualization/quantification system (Bio-Rad Laboratories; Hercules, CA, USA). Relative band densities were evaluated using the InStat statistical analysis program (GraphPad; San Diego).

### Osteoclastic cell differentiation

Non-adherent cells that were expanded for 8 days were re-seeded at 1.8×10^5^ cells/well in 24-well-plates with α-MEM supplemented with 10% fetal bovine serum, 100 units/ml penicillin, 50 µg/ml streptomycin and 1% glutamine, in the presence of 50 ng/ml M-CSF (R&D Systems), and 3–30 ng/ml RANK-L (Peprotech; Rocky Hill, NJ, USA). When osteoclasts were observed (about 6 days in culture) tartrate resistant acid phosphatase (TRAP) staining of osteoclasts was performed using a leukocyte acid phosphatase system (Sigma-Aldrich) performed according to the manufacturer's protocol. Osteoclasts per area were counted. Using the same protocol, forskolin (a cAMP activator) and tetrahydrofurfuryl adenine (THFA) (a cAMP inhibitor) (Sigma-Aldrich) were also used to mimic PTH receptor signaling.

### Immunofluorescence and confocal laser scanning microscopy

Cells were fixed in 4% paraformaldehyde, pH 7.2 for 10 min, permeabilized with 0.2% Triton X-100 for 7 min, then incubated with Anti-vinculin (clone Vin11-5) (Sigma-Aldrich) and F-actin distribution was revealed with AlexaFluor-546-Phalloidin from Molecular Probes (Eugene, OR, USA). Cells were imaged with a confocal Zeiss LSM 510, using a X63 (NA1.4) Plan Neofluor objective. To prevent contamination between fluorochromes, each channel was imaged sequentially, using the multi-track recording module, before merging. Z-cut pictures were obtained using Zeiss LSM 510 software.

### Osteoclast Transmigration Assay

The osteoclast transmigration assay was performed as previously described[23]. In brief osteoclasts were seeded on MC3T3-E1 cell layers, treated with control or 0.1–10 nM PTH then fixed. Cells were stained with phalloidin to visualize actin using confocal microscopy. Cells were imaged with a confocal Zeiss LSM 510, using a X63 (NA1.4) Plan Neofluor objective.

### Osteoclast function (resorption pits)

To determine osteoclast resorptive activity, non-adherent cells that were expanded for 8 days were re-seeded at 1.8×10^5^ cells/well into BD BioCoat™ Osteologic™ Discs (BD Biosciences) or ACC (apatite collagen complexes) coverslips [24] in α-MEM supplemented with 10% fetal bovine serum, 100 units/ml penicillin, 50 µg/ml streptomycin and 1% glutamine in the presence of 50 ng/ml M-CSF and 30 ng/ml RANK-L. Once multinucleation was initially observed, cells were treated with vehicle, PTH or calcitonin (Calbiochem, EMD chemicals; Gibbstown, NJ, USA) as a control. When resorption pits were observed (about 6 days in culture), wells/discs were incubated with 10% sodium hypochlorite for 5 minutes to remove the osteoclasts and wells/discs were rinsed with water and allowed to dry. The resorption pit area was calculated using Image-Pro Plus software (Media Cybernetics; Bethesda, MD, USA) and normalized to the total area. Experiments were performed in duplicate, and two different areas were averaged per well. ACCs were prepared using the method previously described.[24]

### Wright-Giemsa Stain

*Ex vivo* cell amplification was performed as described above. Non-adherent cells from day 4 *ex vivo* cultures or freshly extracted bone marrow were diluted in PBS with 2% bovine serum albumin. Cells were placed in a cytospin apparatus (Thermo Fischer Scientific; Waltham, MA, USA) and centrifuged for 10 minutes at 600 rpm. Cells were stained using the Hema-tek automated slide-stainer (Miles; Elkhart, IN, USA). One hundred cells were scored per slide. Scoring was performed in duplicate.

### Statistical Analyses

All experiments were repeated a minimum of two times in duplicate. Student's *t-*test or ANOVA for independent analyses were performed using the GraphPad InStat Software Program (GraphPad Inc., San Diego, CA, USA). The value of *p*<0.05 was considered significant.

## Results

### PTH expands non-adherent and adherent cells *ex vivo*

An *ex vivo* hematopoietic amplification system was performed to elucidate the mechanisms of PTH action on cells of the hematopoietic lineage. Bone marrow cells were isolated and treated with a single application of PTH, Flt-3L or a combination of both. Flt-3L is produced by several cell types, including stromal cells, and is important for HSC expansion, macrophage survival and development [21], [25].

**Figure 1:**
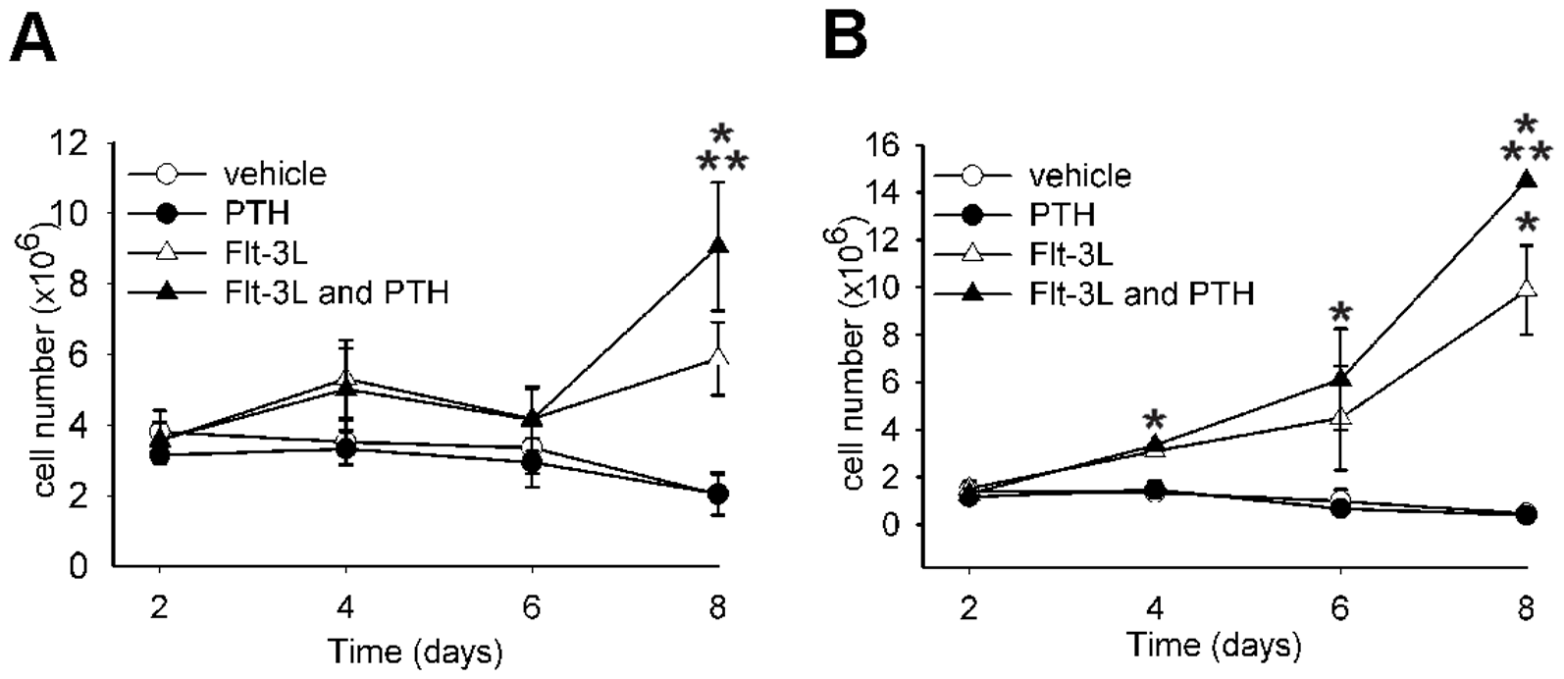
PTH augments Flt-3L cell expansion. Whole bone marrow was isolated from C57B6 mice and seeded at 1.8×10^5^ cells/cm^2^ and treated with vehicle, Flt-3L (100 ng/ml), PTH (10 nM), or a combination of Flt-3L and PTH. Non-adherent (A), and adherent (B), cell populations were harvested at days 2, 4, 6, and 8, then enumerated using trypan blue exclusion. Data shown are mean ± SEM of 2 experiments performed in duplicate. Error bars are present on all data points. * *p*<0.05 versus vehicle ** p<0.05 versus all other groups.

As expected, Flt-3L increased non-adherent cell numbers over an 8-day period (Figure 1A) [21]. When the bone marrow cells were treated with PTH alone, the non-adherent cell pool was not amplified. Interestingly, when PTH was added in conjunction with Flt-3L, there was an additive increase in non-adherent cell numbers compared to Flt-3L alone at day 8, suggesting that PTH selectively targeted the Flt-3L responsive population.

In the adherent cell population, Flt-3L increased the cell numbers starting at day 4 of amplification compared to vehicle (control) (Figure 1B). A single PTH treatment administered to fresh bone marrow cultures did not increase adherent cell numbers when compared to control cells. Similar to non-adherent cells, PTH in combination with Flt-3L significantly increased cell numbers above those of Flt-3L alone at day 8 of amplification, reaffirming that PTH effects are additive in the context of Flt-3L. We observed an increase in adherent hematopoietic cells with Flt-3L treatment (CD11b^+^ and CD45^+^ cells, data not shown). However, the increase in cell numbers could also be due to an increase in the stromal cell population.

### Indirect effect of the PTH on hematopoietic cells expansion

**Figure 2:**
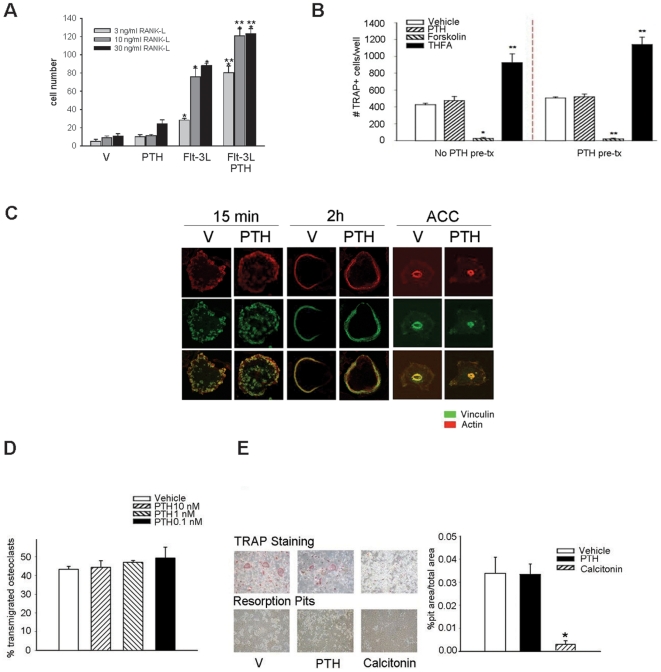
Lack of direct effect of PTH on cells of the osteoclast lineage. (A) Non-adherent cells expanded in the presence or absence of Flt-3L (100 ng/ml) or PTH (10 nM), a combination of both, or vehicle (control), were counted and plated at equal numbers and allowed to differentiate in osteoclastogenic media, then induced via RANK-L (0–30 ng/ml) and M-CSF (50 ng/ml). Five days later, multi-nucleated TRAP^+^ cells were counted. Data are mean ± SEM of 2 experiments performed in duplicate. * *p*<0.05 versus respective vehicle or PTH alone ** *p*<0.05 versus all other groups in their respective RANK-L concentrations. The 0 ng/ml RANK-L resulted in no osteoclasts; therefore, the data was not plotted. (B) Cells were expanded in Flt-3L (5 ng/ml) media for 8 days with or without 10 nM PTH. Cells were counted and plated at equal numbers, then induced via RANK-L (30 ng/ml) and M-CSF (50 ng/ml) to differentiate with additional treatments of PTH, forskolin or tetrahydrofuryladenine (THFA). Multinucleated TRAP^+^ cells were enumerated 5 days later. Data are mean ± SEM of 2 experiments performed in duplicate. *p<0.05 and **p<0.01 versus vehicle. (C) Visualization of the cytoskeleton of actin, by confocal microscopy in mature osteoclasts seeded on coverslips or ACC and stained for actin and vinculin at different time points. All images are the same magnification. (D) Osteoclast transmigration assay: osteoclasts were seeded on MC3T3-E1 cell layers, treated with 0–10 nM PTH then fixed. Cells were stained with phalloidin to visualize actin under confocal microscopy. Data are mean ± SEM of number of osteoclasts that transmigrated compared to the total number of osteoclasts. Experiments were performed a minimum of 3 times. * *p*<0.05 versus vehicle or PTH. (E) Osteoclast functional assay: Cells were expanded in the presence of Flt-3L (100 ng/ml). At day 8, they were seeded onto ACC (TRAP staining) or osteologic disks (resorption pit assay) and induced to differentiate in the presence of 50 ng/ml M-CSF and 30 ng/ml RANK-L. When osteoclasts started to form, PTH, calcitonin or vehicle (control) were added to the medium. Data are mean ± SEM of the area of the pit divided by the total area. Experiments were performed a minimum of 3 times in duplicate. * *p*<0.05 versus vehicle.

Previous reports have demonstrated that cells other than osteoblasts have PTH receptors [26]. To determine if PTH has a direct effect on cells of the hematopoietic lineage, bone marrow cells were treated with PTH and evaluated for their ability to undergo osteoclastic differentiation. Whole bone marrow cells were plated and treated with Flt-3L, PTH, or Flt-3L plus PTH at day 0. At day 8, non-adherent cells were re-plated and cultured in the presence of M-CSF and RANKL (0–30 ng/ml) to induce osteoclast differentiation. TRAP+ cells were enumerated after 6 days of culture, and revealed that Flt-3L and Flt-3L combined with PTH increased osteoclast numbers compared with vehicle treated cells (Figure 2A). PTH alone did not alter osteoclast numbers; however, PTH augmented the Flt-3L effect (Figure 2A). Similarly, pre-treating bone marrow cells with PTH, M-CSF and RANKL did not alter osteoclast differentiation versus no PTH treatment (Figure 2B). Furthermore, the PTH signaling agonist forskolin (FSK) decreased osteoclastogenesis as compared to control, while the tetrahydrofuryladenine (THFA), a cAMP inhibitor, increased osteoclast numbers. This suggests that the cAMP pathway has the ability to directly modulate osteoclastogenesis but in an opposite manner than would be consistent with PTH receptor signaling.

### PTH has no direct effect on osteoclasts and their precursors

Osteoclasts are highly polarized cells and exhibit several features, such as podosomes, when they are spread on glass whereas, when cultured on apatite mineral (ACC), they exhibit another actin-rich structure, the sealing zone, which seals off the resorption area [23], [27]. To determine whether PTH directly altered osteoclast spreading, mature osteoclasts were plated on glass or apatite mineral (ACC) and treated for 15 mins or 2 hours with PTH (Figure 2C). Confocal imaging of vinculin (in green) and F-actin (in red), demonstrated no direct effect of PTH on podosome clusters after 15 min or on podosome belts after 2 hours (Figure 2C). Similarly, the sealing zone formation was not altered when osteoclasts spread on ACC were treated with PTH (Figure 2C). Osteoclasts are also highly migratory cells and able to transmigrate (23). The impact of PTH on osteoclast transmigration was analyzed. Osteoclasts were seeded on MC3T3-E1 cell layers, treated with PTH (0.1–10 nM) or vehicle control then fixed 4 or 12 h after treatment and stained with phalloidin to visualize actin using confocal microscopy. Multinucleated osteoclasts transmigrated through confluent layers of osteoblastic cells in a similar manner whether they were treated with PTH or vehicle control (Figure 2D). Moreover, when osteoclastic cells were treated with PTH there was no alteration in osteoclast resorption capacity, as measured by quantification of resorption pits on a mineralized substrate (Figure 2E). These experiments suggest that PTH does not act directly on hematopoietic cells destined to the osteoclast lineage.

### PTH in combination with Flt-3L decreases cell apoptosis

**Figure 3:**
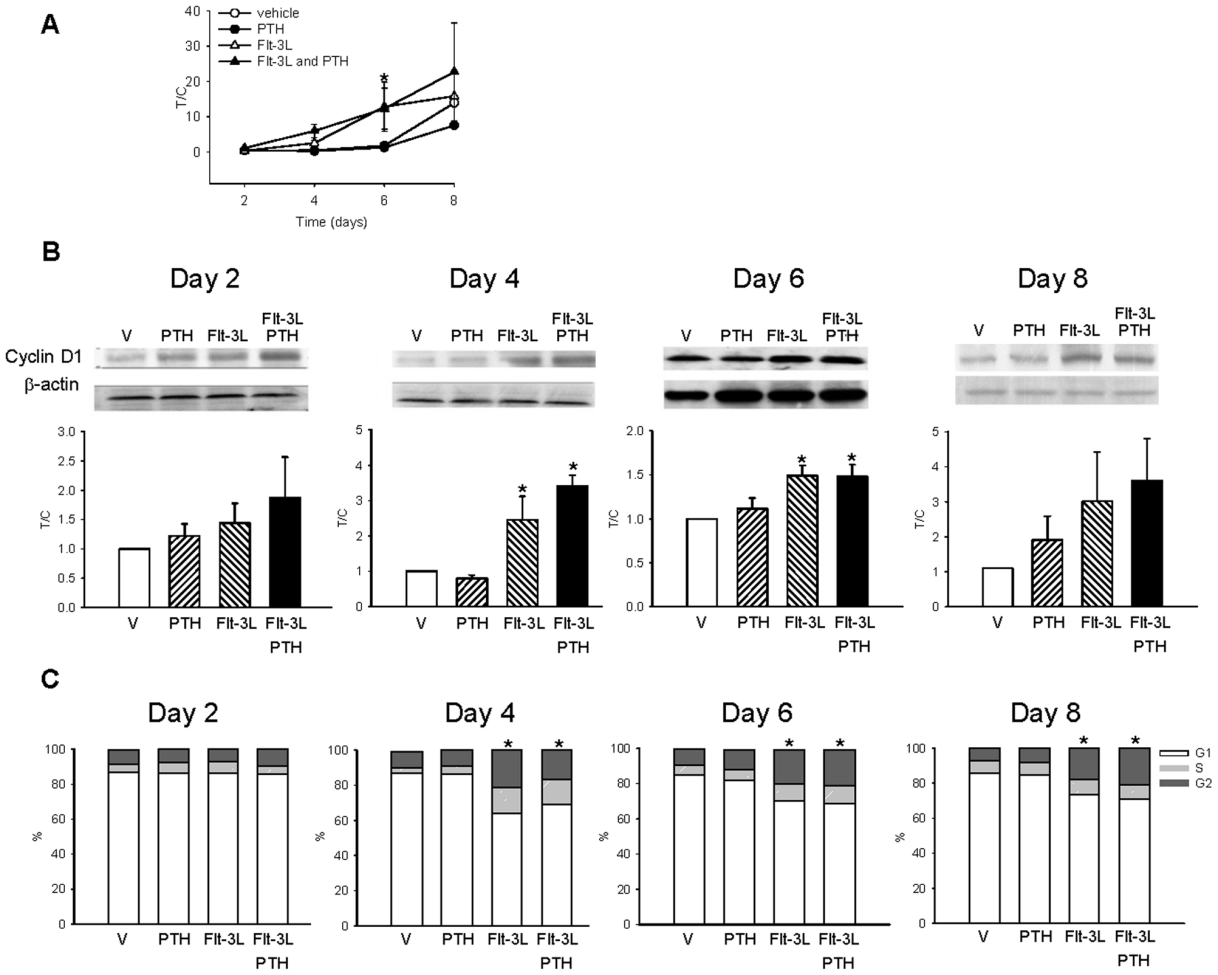
Increased cell proliferation by Flt-3L but not PTH. Whole bone marrow was isolated from C57B6 mice and seeded at 1.8×10^5^ cells/cm^2^ in the presence or absence of Flt-3L (100 ng/ml), PTH (10 nM), a combination of both, or vehicle only control. (A) cyclin D1 mRNA levels of non-adherent cells at days 2, 4, 6, and 8 as determined by real-time PCR. Data are mean ± SEM of at least 3 experiments, performed in duplicate, normalized to GAPDH, and represented as treatment over control (T/C). *p<0.05 versus vehicle. (B) Representative western blot analyses of cyclin D1 and β-actin, and graphs of cyclin D1 protein normalized for β-actin in *ex vivo* cultures at days 2, 4, 6 and 8. Data are mean ± SEM of 3 experiments performed in duplicate, and represented as treatment over control (T/C). * *p*<0.05 vs. vehicle. (C) Flow cytometric analyses of cell cycle *ex vivo*. Graphs represent the percentage of non-adherent cells stained for propidium iodide at days 2, 4, 6 and 8 to demonstrate G1, S and G2 phases. Experiments were performed 4 times in duplicate. *p<0.05 versus vehicle for G1, S and G2 phases.

Since PTH combined with Flt3-L increased hematopoietic cell amplification, the implication of PTH and Flt-3L in cell proliferation and apoptosis was investigated. The mRNA expression and protein levels of cyclin D1 in the non-adherent cell populations were analyzed at days 2, 4, 6 and 8. Cyclin D1 mRNA expression (Figure 3A) and protein levels (Figure 3B) were increased with Flt-3L but there was no significant additive effect with PTH treatment. To further analyze the increase in cell numbers observed in the Flt-3L plus PTH group, cell cycle analysis was performed at days 2, 4, 6 and 8. Flow cytometric analyses demonstrated an increase in G1, S (DNA synthesis) and G2 phases at days 4, 6 and 8; with Flt-3L; however, there was no statistical difference in cell cycle analyses when Flt-3L was compared to the combination PTH plus Flt-3L (Figure 3C).

**Figure 4:**
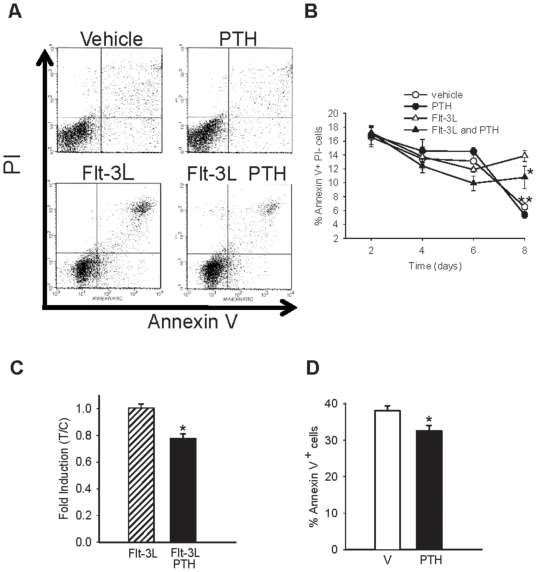
PTH decreased cell apoptosis in a Flt-3L expanded population. Whole bone marrow was isolated from wild-type mice and seeded at 1.8×10^5^ cells/cm^2^ in the presence or absence of Flt-3L (100 ng/ml), PTH (10 nM), a combination of both, or vehicle only, (A) Flow cytometric analyses of Annexin V^+^ Propidium Iodide^-^ (early apoptosis) cells performed on non-adherent cells. Representative Annexin V histogram from day 8. (B) Graph of the fold induction for percentage of Annexin V^+^ cells, (lower right quadrant from histograms represented in A) *p<0.05 versus Flt-3L, ** *p*<0.01 for vehicle and PTH versus Flt-3L. (C) Graph of the fold induction of active caspase 3^+^ cells. Data are mean ± SEM of 4 experiments performed in duplicate. *p<0.05 versus Flt-3L. (D) Four-day-old wild-type C57B6 mice (n≥9/group) were treated daily with 50 µg/kg PTH or vehicle for 3 weeks. Bone marrow was isolated and flow cytometric analyses of Annexin V^+^ cells were performed. Graph of the percentage of Annexin V^+^ cells, *p<0.05 versus vehicle.

Concomitantly, to determine if the increase in cell numbers observed through the combination of PTH plus Flt-3L was due to alterations in cell apoptosis, Annexin V^+^ Propidium Iodide^-^ cells were analyzed by flow cytometry at days 2, 4, 6 and 8. Flow cytometric analyses at day 8 showed a decrease in Annexin V^+^ Propidium Iodide^-^, representing early apoptosis in cells treated with Flt-3L plus PTH compared to those treated with Flt-3L alone (Figures 4 A & B). To further validate the differences observed at day 8 with Flt-3L and the combination of PTH plus Flt-3L, flow cytometric analyses for activated caspase 3 in these two treatment groups were performed. At day 8, there was a statistical decrease in the percentage of active caspase 3 in the group treated with PTH plus Flt-3L compared to the group treated with Flt3-L only (Figure 4C).

To determine if PTH had the ability to decrease cell apoptosis *in vivo*, flow cytometric analyses for Annexin V^+^ cells were performed. Wildtype C57B6 mice received 50 µg/kg of PTH or vehicle daily for 3 weeks as previously described [20]. Bone marrow cells from PTH treated animals had a reduced percentage of Annexin V^+^ early apoptotic cells compared to vehicle treated control mice (Figure 4D). All together, the data suggest a role for PTH in the survival of bone marrow cells *in vivo* and also *ex vivo* when acting in conjunction with Flt-3L, an important ligand in hematopoiesis.

### PTH did not alter the Flt-3L amplified population

**Figure 5:**
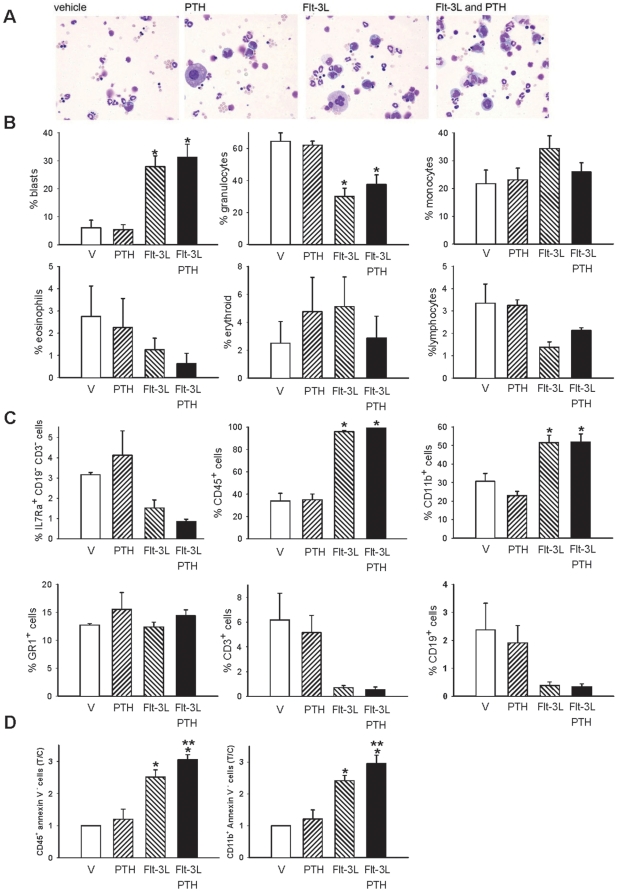
Flt-3L increased blasts, CD45^+^ and CD11b^+^ cells. Whole bone marrow was isolated from wild-type mice and seeded at 1.8×10^5^ cells/cm^2^ in the presence or absence of Flt-3L (100 ng/ml) or PTH (10 nM), a combination of both, or vehicle (control). (A) Representative Wright-Giemsa stain 4 days after cells were seeded and treated once with vehicle, PTH, Flt-3L or the combination of PTH and Flt-3L (40X magnification). (B) Graphs representing the percentage of blasts, granulocytes, monocytes, eosinophils, erythroid cells and lymphocytes that were scored in Wright-Giemsa stained cells at day 4. Data are mean ± SEM of 4 experiments performed in duplicate, *p<0.05 versus vehicle. (C) Graphs representing flow cytometric analyses of the percentage of IL7Ra^+^ CD19^−^ CD3^−^, CD45^+^, CD11b^+^, GR1^+^, CD3^+^ and CD19^+^ cells in the non-adherent cell populations at day 8. Data are mean ± SEM of 4 experiments performed in duplicate, *p<0.05 versus vehicle. (D) Graphs representing flow cytometric analyses of the fold induction (T/C) of CD45^+^ Annexin V^−^ cells and CD11b^+^ Annexin V^−^ cells in the non-adherent cell population at day 8. Data are mean ± SEM of 2 experiments performed in triplicate, *p<0.05 versus vehicle, ** *p*≤0.05 versus all other groups.

Flt-3L is known for its ability to enrich the myeloid cell population as evidenced by an increase in CD11b^+^ cells [21]. To evaluate the phenotype of the non-adherent cells responsive to PTH in our system, a morphologic analysis was performed on Wright-Giemsa stained preparations of the non-adherent cell population at day 4 of culture (Figure 5A). Blast cell numbers were increased in the presence of Flt-3L, whereas granulocyte numbers were decreased at day 4 in culture. The number of monocytes, eosinophils, lymphocytes and erythroid cells in Flt-3L treated groups were not different compared to vehicle and PTH treated groups (Figure 5B). PTH did not alter the Flt-3L effects on cell populations at day 4 in culture (Figure 5B). To further determine the phenotype of the Flt-3L and the Flt-3L in combination with PTH groups, flow cytometric analyses for IL7Ra^+^ CD19^−^ CD3^−^ (lymphoid progenitor), CD45^+^(myeloid), CD11b^+^(monocyte/macrophage), GR1^+^(granulocyte), CD3^+^ (T-cell), and CD19^+^ (B-cell) cells were performed in the non-adherent cell populations at day 8 in culture. Flt-3L significantly increased the CD45^+^ and CD11b^+^ cells in the non-adherent cell population. Flt-3L did not alter the IL7Ra^+^ CD19^−^ CD3^−^, GR1^+^, CD3^+^ nor the CD19^+^. PTH had no specific effect on any of the markers in the non-adherent cell population (Figure 5C). When CD11b+ cells and CD45+ cells were also gated for Annexin V- cells (live cells), there was an increase in cell numbers with Flt-3L. However, there was further increase when PTH was added in combination with Flt-3L (Figure 5D), suggesting that PTH decreases apoptosis of myeloid cell populations.

### PTH expansion is mediated by IL-6 *ex vivo*

**Figure 6:**
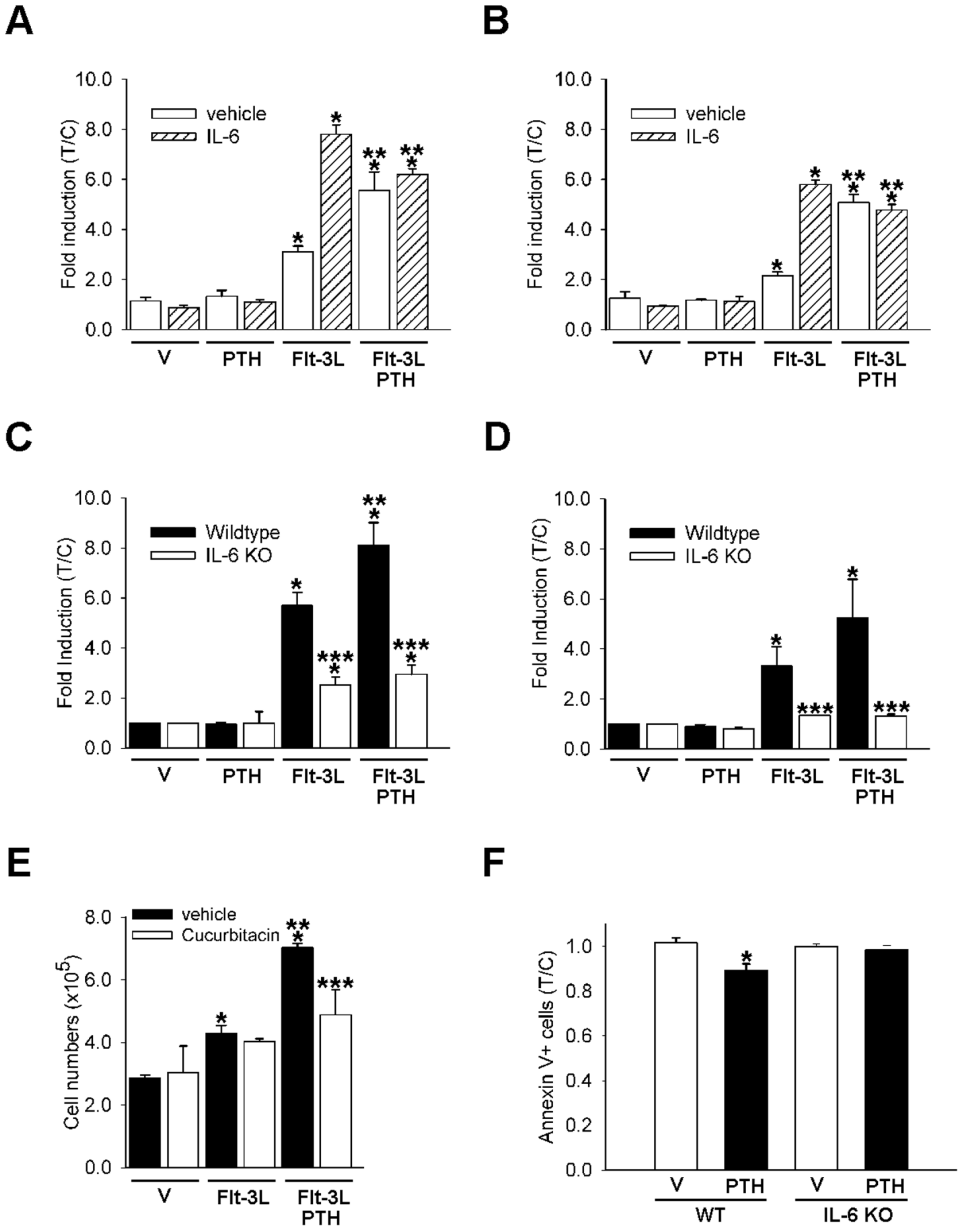
IL-6 mediates the *ex vivo* and *in vivo* PTH effects. (A–B) Whole bone marrow was isolated from wild-type (WT) mice and seeded at 1.8×10^5^ cells/cm^2^ in the presence or absence of Flt-3L (100 ng/ml) or PTH (10 nM), a combination of both, or vehicle only control, with and without IL-6 (10 ng/ml). Non-adherent (A), and adherent (B), cells were harvested and enumerated using trypan blue exclusion at day 8. Data are mean ± SEM of 2 experiments performed in duplicate *p<0.05 versus vehicle/vehicle, ** p<0.05 versus vehicle/Flt-3L. (C–D) Whole bone marrow was isolated from wild-type or IL-6 deficient mice (IL-6 KO) and seeded at 1.8×10^5^ cells/cm^2^ in the presence of Flt-3L (100 ng/ml), PTH (10 nM), a combination of PTH and Flt-3L, or vehicle only control. Non-adherent (C) and adherent (D) cells were harvested and enumerated using trypan blue exclusion at day 8 of culture. Data are mean ± SEM of 2 experiments performed in duplicate. *p<0.05 versus vehicle (wild-type cells) **p<0.05 versus Flt-3L (wild-type cells) ***p<0.05 IL6-KO cells versus wild-type cells of the respective treatment group. Whole bone marrow was isolated from wild-type mice and seeded at 1.8×10^5^ cells/cm^2^ in the presence or absence of Flt-3L (100 ng/ml) or PTH (10 nM), a combination of both. One hour after cells were plated vehicle (control) or a STAT-3 inhibitor, cucurbitacin (20 nM) was added to the culture. Non-adherent cells were harvested and enumerated using trypan blue exclusion at day 8 of culture (E). Data are mean ± SEM, from one of two experiments performed with similar results, *p<0.05 versus vehicle of the respective group, **p<0.05 versus Flt-3L of the respective group, ***p<0.05 vehicle versus cucurbitacin in the combined Flt-3L and PTH groups. (F) Four-day-old wild-type and IL-6-deficient mice (n≥5/group) were treated daily with 50 µg/kg PTH or vehicle for 3 weeks. Bone marrow was isolated and flow cytometric analyses of Annexin V^+^ cells were performed. Fold induction of Annexin V+ cells measured as treatment over vehicle (control) of the respective phenotype. *p<0.05 versus vehicle of the respective phenotype.

PTH is well known to increase the production and secretion of IL-6 [14], a factor which plays an important role in hematopoiesis [28]. To determine if IL-6 is a factor implicated in the *ex vivo* bone marrow amplification observed in the presence of PTH and Flt-3L, bone marrow cells were cultured for 8 days with a single treatment of PTH, Flt-3L or PTH plus Flt-3L in the presence and absence of IL-6 at the time of plating. IL-6 alone did not alter cell amplification (Figure 6). Interestingly, IL-6 had an additive effect on the Flt-3L amplification of both cell populations (Figure 6A–B), which was similar to that seen with PTH in the non-adherent and adherent cell populations.

To further validate the IL-6 impact on hematopoietic cell expansion, bone marrow cells derived from wild-type and IL-6 deficient mice were isolated and cultured with a single treatment of Flt-3L, PTH or combined treatment for a period of 8 days. At day 8, adherent and non-adherent cells were enumerated. Flt-3L increased both populations in cells derived from the wild-type bone marrow and the combined treatment had an additive effect compared Flt-3L alone (Figure 6C–D). Interestingly, the amplification of both populations, with Flt-3L alone or combined with PTH was lower in the bone marrow cultures derived from the IL-6 deficient mice. More precisely, no added amplification with PTH was observed for the non-adherent population (Figure 6C). While a slight increase was noticed after Flt-3L alone or in combination with PTH in the adherent cell populations, no additive effect was observed with the addition of PTH to Flt-3L (Figure 6D). Moreover, when IL-6 signaling was blocked in the non-adherent cell population by cucurbitacin (a STAT-3 inhibitor)[29], there was a decrease in the ability of PTH to increase cell numbers in the presence of Flt-3L (Figure 6E). PTH decreased cell apoptosis in vivo as measured by a decrease in the percentage of Annexin V+ cells (Figure 4D). To determine if IL-6 mediates the PTH ability to decrease cell apoptosis *in vivo*, flow cytometric analyses for Annexin V^+^ cells was performed. Wildtype and IL-6 deficient mice received 50 µg/kg of PTH or vehicle daily for 3 weeks. Bone marrow cells from PTH treated animals had a reduced percentage of Annexin V^+^ early apoptotic cells compared to vehicle treated mice. In contrast, PTH failed to decrease the percentage of apoptotic cells in IL-6 deficient mice (Figure 6F). These data implicate IL-6 in the PTH pathway that mediates hematopoietic cell maintenance and amplification.

### PTH fails to increase HPCs in IL-6 deficient mice

**Figure 7:**
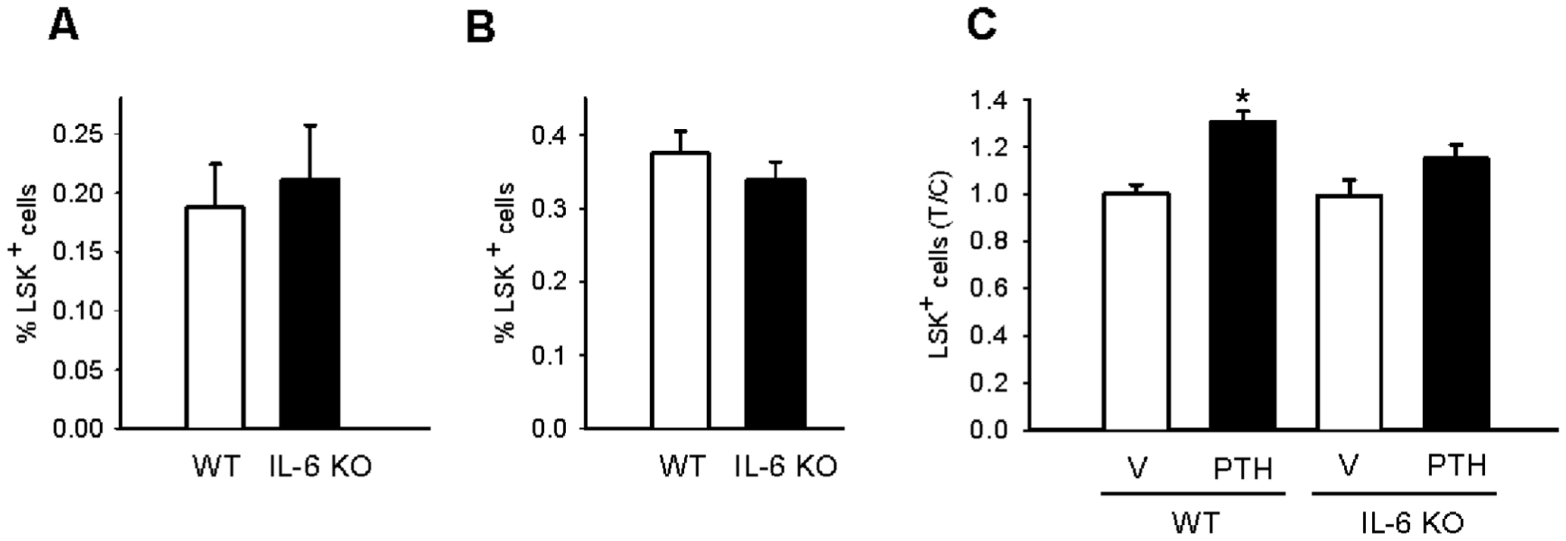
PTH failed to increase LSK cells in IL-6 deficient mice. A) Bone marrow was isolated from four-day-old wild-type (WT) and IL-6-deficient mice IL-6 KO) (n≥9/group) and flow cytometric analyses of Lin^-^ Sca-1^+^ c-Kit^+^ (LSK) cells were performed. Graph represents the percentage of LSK cells out of total bone marrow cells. B) Bone marrow was isolated from 26-day-old wild-type and IL-6 deficient mice (n≥7/group) and flow cytometric analyses of Lin^-^ Sca-1^+^ c-Kit^+^ (LSK) cells were performed. Graph represents the percentage of LSK cells per total bone marrow cells. C) Four-day-old wild-type and IL-6-deficient mice (n≥6/group) were treated daily with 50 µg/kg PTH or vehicle for 3 weeks. Bone marrow was isolated and flow cytometric analyses of Lin^-^ Sca-1^+^ c-Kit^+^ (LSK) cells were performed. Fold induction of LSK cells measured as treatment over vehicle (control) of the respective phenotype, *p<0.05 versus wildtype vehicle.

PTH increases the Lin^-^ Sca-1^+^ c-Kit^+^ (LSK) population of hematopoietic progenitor cells *in vivo* [5]. Flow cytometric analyses were performed from bone marrow cells of wildtype and IL-6 deficient mice at baseline day 4 and day 26 to determine if there were any inherent differences prior to PTH treatment. There were no baseline or day 26 differences in LSK cells (Figure 7A,B). Four-day-old-mice received 50 µg/kg of PTH or vehicle daily for 3 weeks as previously described to determine if PTH increased hematopoietic progenitor cells in a skeletally responsive animal model [20]. Forty-eight hours after the last PTH injection, bone marrow cells were isolated and flow cytometric analysis was performed to measure LSK cells. PTH significantly increased the percentage of LSK cells after 3 weeks of intermittent PTH administration in wild-type mice but not IL-6-deficient mice (Figure 7C).

## Discussion

PTH increases cells of the hematopoietic lineage, including hematopoietic progenitor cells [5]. The present study revealed that PTH increased cells of the hematopoietic lineage by indirectly decreasing hematopoietic cell apoptosis. Furthermore, IL-6 mediated the PTH effect on cells of the hematopoietic lineage *in vivo* and *ex vivo*.

In the *ex vivo* expansion amplification model described in this paper, Flt-3L had direct effects in cells of the hematopoietic lineage, which would be expected since the receptor for Flt-3L is expressed in these cells and not in stromal cells [30]. Although Flt-3L alone is sufficient for hematopoietic cell amplification *in vitro* [21], it acts synergistically with other factors including IL-6 leading to an increase in cell proliferation [31]. In the present study PTH augmented the Flt-3L increase in hematopoietic cell numbers *ex vivo*. Moreover, IL-6, a well known downstream target induced in osteoblasts by PTH [14] was a mediator of this effect. The ability of PTH to increase progenitor cells *in vivo* through IL-6 may explain the mechanism by which PTH is increasing hematopoietic progenitor cells. These results may provide insights to the correlation of CD34^+^ progenitor cells and PTH levels in patients with hyperparathyroidism and the increase in regeneration seen with PTH treatment after myocardial infarction [32], [33].

The role of PTH in apoptosis has been extensively documented in cells that express the PTH-1 receptor such as cells of the osteoblastic lineage [34], [35]. The current experiments demonstrate an indirect anti-apoptotic effect of PTH on cells of the hematopoietic lineage. IL-6 has an important role in cell survival and prevents apoptosis of several hematopoietic cells including T-cells and early plasma cells [15], [36], [37]. Data from the present study suggests that PTH-induced stromal cell-derived IL-6 promotes hematopoietic cell survival.

PTH was capable of expanding the adherent cell population but only in conjunction with Flt-3L, suggesting that the PTH effect depends on a stimulus from cells of the hematopoietic lineage. The increase in adherent cell numbers with Flt-3L and the combination, Flt-3L plus PTH, could reflect an increase in adherent hematopoietic cells, an increase in the stromal cell population or an increase in both. Bone marrow adherent cells are a heterogeneous population, where it is estimated that 10–20% are mesenchymal stem cells and approximately 80% are lymphohematopoietic cells [38]. During normal macrophage expansion there is also an increase in monocytes in the adherent cell population, and Flt-3L has been shown to increase the adherent monocytic population [39], [40]. Therefore, the increase in adherent cell numbers may be due to an increase in adherent hematopoietic cells, more specifically the monocyte/macrophage population. Such an increase in may signify a beneficial action of PTH given that macrophages were recently reported to promote osteoblastic differentiation [41]. In our model system, the indirect effect of PTH on stromal cells cannot be ruled out, particularly since the indirect effect of PTH on cells of the osteoblastic lineage has been demonstrated [42]. Data from our laboratory corroborate this finding where it was demonstrated that under compromised osteoclast differentiation, anabolic actions of PTH were blocked [20], [43]. Taken together, PTH acts on stromal cells which, in turn, signal to hematopoietic cells. Data from the present study suggest that these hematopoietic cells then signal back to the stromal cells. This is an area worthy of future investigation.

The PTH effect on the Flt-3L stimulated cells was mediated largely by IL-6. IL-6 mimicked the PTH additive effect with Flt-3L in both the non-adherent and the adherent cell populations. Moreover, when bone marrow cells from IL-6 deficient-mice were treated with PTH, they failed to have the additive effect on the Flt-3L expanded cells. In addition to being a mediator of hematopoiesis as previously described [44], IL-6 was shown here to be responsible for hematopoietic cell expansion *ex vivo* and *in vivo*. The additive effect of PTH in combination with Flt-3L on cells of the hematopoietic lineage can be explained by the synergism of IL-6 with Flt-3L, which results in proliferation of primitive lymphohematopoietic progenitor cells [45]. Another possible mechanism by which the PTH increase in IL-6 may be influencing cells of the hematopoietic lineage is by increasing Flt-3L expression, given that IL-6 in conjunction with its receptor, IL-6R, has the ability to enhance Flt-3L expression in NIH3T3 cells [46].

The role of osteoblasts in support of hematopoiesis has been established and the reverse role of hematopoietic cell support of osteoblasts has also been described [47], [48]. In the present study, the adherent cell population consists in part of pre-osteoblastic mesenchymal cells. It is likely that PTH induced stromal derived IL-6 which then acted on cells of the hematopoietic lineage. The IL-6 receptor is expressed in cells of the hematopoietic lineage and it is widely accepted that IL-6 acts directly on osteoclasts whereas the IL-6R is weakly expressed or even absent in stromal/osteoblastic cells [15], [49]. Thus, the direct effect of IL-6 on osteoblasts would only be possible if soluble IL-6 receptor was added *in vitro* [50]. In the experiments presented here, the direct role of IL-6 on stromal cells is improbable since IL-6 treatment alone did not increase cell numbers. Moreover, IL-6 deficient mice have a defect in hematopoiesis that is attributed to the lack of IL-6 in the stromal cell compartment [51]. Therefore, IL-6 does not directly act on stromal cells but instead targets the hematopoietic cells. PTH impacts the increase in hematopoietic progenitor cells indirectly via its regulation of IL-6.

Three weeks of PTH treatment in wildtype mice increased the Lin^-^Sca-1^+^c-Kit^+^ population of hematopoietic progenitor cells *in vivo*. In contrast, PTH failed to increase hematopoietic progenitor cells in age matched IL-6 deficient mice. IL-6 enhances proliferation of HPCs [44]. Mice overexpressing IL-6 and sIL6R show massive extramedullary hematopoiesis in their spleen and liver [52]. Given that PTH increases IL-6 expression, the importance of PTH in hematopoiesis is significant. Equally significant is the failure of PTH to increase HPCs in IL-6-deficient-mice. Calvi et al. [5] reported that IL-6 was upregulated in PTH1R-overexpressing-mice, with increased hematopoietic progenitor cells but there was no definitive link made with PTH, hematopoiesis, and IL-6 in that study. The present study provides a mechanistic role for PTH in hematopoiesis.

**Figure 8:**
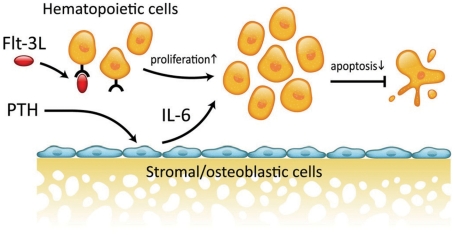
Working Model: IL-6 mediates the PTH anti-apoptotic effect on cells of the hematopoietic lineage. PTH induces IL-6 expression in stromal cells. Flt-3L increases hematopoietic cell proliferation. IL-6 acts in conjunction with Flt-3L reducing apoptosis in the hematopoietic cell compartment.

In summary, PTH increases hematopoietic cells *ex vivo* via an inhibition of apoptosis in Flt-3L responsive cells. PTH indirectly increases hematopoietic progenitor cells and does not directly affect osteoclast lineage cells. Stromal cell derived IL-6 in conjunction with Flt-3L mediates the PTH activation of hematopoietic cells (Figure 8).

## References

[1] Philbrick WM, Dreyer BE, Nakchbandi IA, Karaplis AC (1998). Parathyroid hormone-related protein is required for tooth eruption.. Proc Natl Acad Sci U S A.

[2] Bryden AA, Hoyland JA, Freemont AJ, Clarke NW, George NJ (2002). Parathyroid hormone related peptide and receptor expression in paired primary prostate cancer and bone metastases.. Br J Cancer.

[3] Wysolmerski JJ, Stewart AF, Kovacs CS, Bilezikian J, Raisz LG, Rodan GA (2008). Physiological Actions of parathyroid hormone and parathyroid hormone related protein.. Principles of Bone Biology.

[4] Adams GB, Martin RP, Alley IR, Chabner KT, Cohen KS (2007). Therapeutic targeting of a stem cell niche.. Nat Biotechnol.

[5] Calvi LM, Adams GB, Weibrecht KW, Weber JM, Olson DP (2003). Osteoblastic cells regulate the haematopoietic stem cell niche.. Nature.

[6] Ballen KK, Shpall EJ, Avigan D, Yeap BY, Fisher DC (2007). Phase I trial of parathyroid hormone to facilitate stem cell mobilization.. Biol Blood Marrow Transplant.

[7] Lee SK, Lorenzo JA (1999). Parathyroid hormone stimulates TRANCE and inhibits osteoprotegerin messenger ribonucleic acid expression in murine bone marrow cultures: correlation with osteoclast-like cell formation.. Endocrinology.

[8] Gay CV (1991). Avian osteoclasts.. Calcif Tissue Int.

[9] Gay CV, Zheng B, Gilman VR (2003). Co-detection of PTH/PTHrP receptor and tartrate resistant acid phosphatase in osteoclasts.. J Cell Biochem.

[10] Komarova SV (2005). Mathematical model of paracrine interactions between osteoclasts and osteoblasts predicts anabolic action of parathyroid hormone on bone.. Endocrinology.

[11] Terauchi M, Li JY, Bedi B, Baek KH, Tawfeek H (2009). T lymphocytes amplify the anabolic activity of parathyroid hormone through Wnt10b signaling.. Cell Metab.

[12] McCauley LK, Rosol TJ, Merryman JI, Capen CC (1992). Parathyroid hormone-related protein binding to human T-cell lymphotropic virus type I-infected lymphocytes.. Endocrinology.

[13] Hory BG, Roussanne MC, Rostand S, Bourdeau A, Drueke TB (2000). Absence of response to human parathyroid hormone in athymic mice grafted with human parathyroid adenoma, hyperplasia or parathyroid cells maintained in culture.. J Endocrinol Invest.

[14] Greenfield EM, Gornik SA, Horowitz MC, Donahue HJ, Shaw SM (1993). Regulation of cytokine expression in osteoblasts by parathyroid hormone: rapid stimulation of interleukin-6 and leukemia inhibitory factor mRNA.. J Bone Miner Res.

[15] Kishimoto T, Akira S, Narazaki M, Taga T (1995). Interleukin-6 family of cytokines and gp130.. Blood.

[16] Michalevicz R, Lifshitz D, Revel M (1989). Interferon beta 2/interleukin-6 and interleukin-3 synergize in stimulating proliferation of human early hematopoietic progenitor cells.. Scanning Microsc.

[17] O'Brien CA, Jilka RL, Fu Q, Stewart S, Weinstein RS (2005). IL-6 is not required for parathyroid hormone stimulation of RANKL expression, osteoclast formation, and bone loss in mice.. Am J Physiol Endocrinol Metab.

[18] Poli V, Balena R, Fattori E, Markatos A, Yamamoto M (1994). Interleukin-6 deficient mice are protected from bone loss caused by estrogen depletion.. Embo J.

[19] Kopf M, Baumann H, Freer G, Freudenberg M, Lamers M (1994). Impaired immune and acute-phase responses in interleukin-6-deficient mice.. Nature.

[20] Demiralp B, Chen HL, Koh AJ, Keller ET, McCauley LK (2002). Anabolic actions of parathyroid hormone during bone growth are dependent on c-fos.. Endocrinology.

[21] Servet-Delprat C, Arnaud S, Jurdic P, Nataf S, Grasset MF (2002). Flt3+ macrophage precursors commit sequentially to osteoclasts, dendritic cells and microglia.. BMC Immunol.

[22] Livak KJ, Schmittgen TD (2001). Analysis of relative gene expression data using real-time quantitative PCR and the 2(-Delta Delta C(T)) Method.. Methods.

[23] Saltel F, Chabadel A, Zhao Y, Lafage-Proust MH, Clezardin P (2006). Transmigration: a new property of mature multinucleated osteoclasts.. J Bone Miner Res.

[24] Shibutani T, Iwanaga H, Imai K, Kitago M, Doi Y (2000). Use of glass slides coated with apatite-collagen complexes for measurement of osteoclastic resorption activity.. J Biomed Mater Res.

[25] Brasel K, McKenna HJ, Morrissey PJ, Charrier K, Morris AE (1996). Hematologic effects of flt3 ligand in vivo in mice.. Blood.

[26] Dempster DW, Hughes-Begos CE, Plavetic-Chee K, Brandao-Burch A, Cosman F (2005). Normal human osteoclasts formed from peripheral blood monocytes express PTH type 1 receptors and are stimulated by PTH in the absence of osteoblasts.. J Cell Biochem.

[27] Jurdic P, Saltel F, Chabadel A, Destaing O (2006). Podosome and sealing zone: specificity of the osteoclast model.. Eur J Cell Biol.

[28] Bernad A, Kopf M, Kulbacki R, Weich N, Koehler G (1994). Interleukin-6 is required in vivo for the regulation of stem cells and committed progenitors of the hematopoietic system.. Immunity.

[29] Blaskovich MA, Sun J, Cantor A, Turkson J, Jove R (2003). Discovery of JSI-124 (cucurbitacin I), a selective Janus kinase/signal transducer and activator of transcription 3 signaling pathway inhibitor with potent antitumor activity against human and murine cancer cells in mice.. Cancer Res.

[30] Drexler HG, Quentmeier H (2004). FLT3: receptor and ligand.. Growth Factors.

[31] Rusten LS, Lyman SD, Veiby OP, Jacobsen SE (1996). The FLT3 ligand is a direct and potent stimulator of the growth of primitive and committed human CD34+ bone marrow progenitor cells in vitro.. Blood.

[32] Brunner S, Theiss HD, Murr A, Negele T, Franz WM (2007). Primary hyperparathyroidism is associated with increased circulating bone marrow-derived progenitor cells.. Am J Physiol Endocrinol Metab.

[33] Zaruba MM, Huber BC, Brunner S, Deindl E, David R (2008). Parathyroid hormone treatment after myocardial infarction promotes cardiac repair by enhanced neovascularization and cell survival.. Cardiovasc Res.

[34] Jilka RL, Weinstein RS, Bellido T, Roberson P, Parfitt AM (1999). Increased bone formation by prevention of osteoblast apoptosis with parathyroid hormone.. J Clin Invest.

[35] Chen HL, Demiralp B, Schneider A, Koh AJ, Silve C (2002). Parathyroid hormone and parathyroid hormone-related protein exert both pro- and anti-apoptotic effects in mesenchymal cells.. J Biol Chem.

[36] Chauhan D, Kharbanda S, Ogata A, Urashima M, Teoh G (1997). Interleukin-6 inhibits Fas-induced apoptosis and stress-activated protein kinase activation in multiple myeloma cells.. Blood.

[37] Kawano MM, Mihara K, Huang N, Tsujimoto T, Kuramoto A (1995). Differentiation of early plasma cells on bone marrow stromal cells requires interleukin-6 for escaping from apoptosis.. Blood.

[38] Phinney DG, Kopen G, Isaacson RL, Prockop DJ (1999). Plastic adherent stromal cells from the bone marrow of commonly used strains of inbred mice: variations in yield, growth, and differentiation.. J Cell Biochem.

[39] Way KJ, Dinh H, Keene MR, White KE, Clanchy FI (2009). The generation and properties of human macrophage populations from hemopoietic stem cells.. J Leukoc Biol.

[40] Lean JM, Fuller K, Chambers TJ (2001). FLT3 ligand can substitute for macrophage colony-stimulating factor in support of osteoclast differentiation and function.. Blood.

[41] Chang MK, Raggatt LJ, Alexander KA, Kuliwaba JS, Fazzalari NL (2008). Osteal tissue macrophages are intercalated throughout human and mouse bone lining tissues and regulate osteoblast function in vitro and in vivo.. J Immunol.

[42] Pettway GJ, Meganck JA, Koh AJ, Keller ET, Goldstein SA (2008). Parathyroid hormone mediates bone growth through the regulation of osteoblast proliferation and differentiation.. Bone.

[43] Koh AJ, Demiralp B, Neiva KG, Hooten J, Nohutcu RM (2005). Cells of the osteoclast lineage as mediators of the anabolic actions of parathyroid hormone in bone.. Endocrinology.

[44] Ikebuchi K, Wong GG, Clark SC, Ihle JN, Hirai Y (1987). Interleukin 6 enhancement of interleukin 3-dependent proliferation of multipotential hemopoietic progenitors.. Proc Natl Acad Sci U S A.

[45] Hirayama F, Lyman SD, Clark SC, Ogawa M (1995). The flt3 ligand supports proliferation of lymphohematopoietic progenitors and early B-lymphoid progenitors.. Blood.

[46] Peters M, Solem F, Goldschmidt J, Schirmacher P, Rose-John S (2001). Interleukin-6 and the soluble interleukin-6 receptor induce stem cell factor and Flt-3L expression in vivo and in vitro.. Exp Hematol.

[47] Taichman RS, Emerson SG (1994). Human osteoblasts support hematopoiesis through the production of granulocyte colony-stimulating factor.. J Exp Med.

[48] Jung Y, Song J, Shiozawa Y, Wang J, Wang Z (2008). Hematopoietic stem cells regulate mesenchymal stromal cell induction into osteoblasts thereby participating in the formation of the stem cell niche.. Stem Cells.

[49] Palmqvist P, Persson E, Conaway HH, Lerner UH (2002). IL-6, leukemia inhibitory factor, and oncostatin M stimulate bone resorption and regulate the expression of receptor activator of NF-kappa B ligand, osteoprotegerin, and receptor activator of NF-kappa B in mouse calvariae.. J Immunol.

[50] Erices A, Conget P, Rojas C, Minguell JJ (2002). Gp130 activation by soluble interleukin-6 receptor/interleukin-6 enhances osteoblastic differentiation of human bone marrow-derived mesenchymal stem cells.. Exp Cell Res.

[51] Rodriguez Mdel C, Bernad A, Aracil M (2004). Interleukin-6 deficiency affects bone marrow stromal precursors, resulting in defective hematopoietic support.. Blood.

[52] Schirmacher P, Peters M, Ciliberto G, Blessing M, Lotz J (1998). Hepatocellular hyperplasia, plasmacytoma formation, and extramedullary hematopoiesis in interleukin (IL)-6/soluble IL-6 receptor double-transgenic mice.. Am J Pathol.

